# Healthcare professional-led interventions on lifestyle modifications for hypertensive patients – a systematic review and meta-analysis

**DOI:** 10.1186/s12875-021-01421-z

**Published:** 2021-04-05

**Authors:** Indre Treciokiene, Maarten Postma, Thang Nguyen, Tanja Fens, Jurgis Petkevicius, Raimondas Kubilius, Jolanta Gulbinovic, Katja Taxis

**Affiliations:** 1 Unit of PharmacoTherapy, -Epidemiology & -Economics, Groningen Research Institute of Pharmacy (GRIP), University of Groningen, Groningen, Netherlands; 2 Pharmacy Center, Faculty of Medicine, Vilnius University, Vilnius, Lithuania; 3 Department of Health Sciences, University of Groningen, University Medical Center Groningen, Groningen, Netherlands; 4 Department of Economics, Econometrics & Finance, Faculty of Economics & Business, University of Groningen, Groningen, Netherlands; 5 Pharmacology & Clinical Pharmacy Department, Can Tho University of Medicine and Pharmacy, Can Tho City, Vietnam; 6 Lithuanian University of Health Sciences, Kaunas, Lithuania

**Keywords:** Hypertension, Blood pressure, Non-pharmacological intervention, Lifestyle, Health care professionals

## Abstract

**Background:**

About 0.9 billion people in the world have hypertension. The mortality due to hypertension increased dramatically over the last decades. Healthcare professionals should support patients with hypertension to modify their lifestyle to decrease blood pressure, but an overview of effective lifestyle interventions is lacking. The aim of this study was to determine whether healthcare professional-led interventions on lifestyle modifications are effective in lowering blood pressure in patients with hypertension.

**Methods:**

A systematic literature review following the PRISMA guidelines was conducted. PubMed, EMBASE and CINAHL databases were searched for randomized control trials (RCTs) of interventions on lifestyle modifications of hypertensive patients which were performed by healthcare professionals (physician, nurse, pharmacist) and which reported blood pressure measurements. Papers were reviewed by two reviewers and analysed using Cochrane software Revman 5.4. In a meta-analysis difference in systolic blood pressure (SBP), diastolic blood pressure (DBP) and the percentage of patients with controlled blood pressure (BP) was analysed.

**Results:**

In total, 34 clinical trials reporting on 22,419 patients (mean age 58.4 years, 49.14% female, 69.9% used antihypertensive medications) were included. The mean difference SBP was − 4.41 mmHg (95% CI, − 5.52to − 3.30) and the mean difference DBP was − 1.66 mmHg (95% CI − 2.44 to − 0.88) in favor of the intervention group vs usual care. Fifty-six percent of patients achieved BP control in the intervention group vs 44% in usual care, OR = 1.87 (95% CI, 1.51 to 2.31).

**Conclusion:**

Healthcare professional-led interventions were effective. Patients achieved almost 5 mmHg decrease of SBP and more patients achieved BP control. The results suggest that efforts are needed for widespread implementation.

**Supplementary Information:**

The online version contains supplementary material available at 10.1186/s12875-021-01421-z.

## Background

Cardiovascular diseases (CVDs) remain the most common cause of death worldwide, causing 17.3 million (31.5%) deaths globally [1]. One of the most important risk factors to develop CVDs is hypertension [1]. Globally WHO reports suggest that 1in 5 adults had raised blood pressure [2]. In the same time period, the prevalence of hypertension among US adults of 20 years of age or older was estimated to be even 34.0% [3]. Death rates due to hypertension have increased worldwide and are associated with high costs [4]. In the U.S.A. the annual national spending on hypertension increased significantly from $58.7 billion to $109.1 billion from 2000 to 2001 to 2012–2013 [5] and is associated with about $131 billion per year in population-level expenditures [6].

Unhealthy diet, physical inactivity and obesity increase the risk of developing hypertension [7]. Lifestyle change is a key component in the cardiovascular risk management and essential in decreasing blood pressure [8–10]. Studies evaluating lifestyle modifications such as weight-reducing diets, regular exercise as well as restricted alcohol and salt intake showed positive effects on blood pressure [11]. In a systematic review, Dickinson et al. assessed the effects of the different lifestyle modifications. Improved diet resulted in a mean reduction of systolic blood pressure (SBP) of − 5.0 mmHg (95% CI, − 7.0 to − 3.1), aerobic exercise − 4.6 mmHg (95% CI, − 7.1 to − 2.0), alcohol restriction - 3.8 mmHg (95% CI, − 6.1 to − 1.4) and sodium restriction − 3.6 mmHg (95% CI, − 4.6 to − 2.5) [12]. Healthcare professionals have an important role in supporting patients in achieving such lifestyle alterations to improve blood pressure control. Previous systematic reviews have shown that pharmacist- [13] and nurse- [14] led interventions can be successful in improving blood pressure control, but those studies did not focus on interventions of lifestyle modification. In another systematic review, it was shown that physician-led interventions result in significant weight losses, but this study did not assess hypertension [15]. An overview of effective lifestyle interventions which can be performed by healthcare professionals is lacking. Therefore, the aim of this study was to determine whether healthcare professional-led interventions on lifestyle modifications are effective in lowering blood pressure in patients with hypertension.

## Methods

A systematic literature review and meta-analysis following the PRISMA guidelines for reporting was performed [16]. A literature search for studies evaluating interventions on lifestyle modifications in patients with hypertension was conducted.

### Inclusion and exclusion criteria

Included studies:

- Were randomized controlled clinical trials (RCTs);
- Involved patients with a diagnosis of hypertension or with an elevated blood pressure above 140/90 mmHg or 130/80 for patients with diabetes [17];
- Evaluated an intervention led by a healthcare professional which consisted of one or more individual consultations on lifestyle modification, health promotion or non-pharmaceutical management, targeting blood pressure.

Excluded studies:

- Were non-English articles;
- Interventions performed in group sessions;
- Consisted of pharmacological interventions only;
- Interventions that included less than 10 patients in either intervention or control group

Studies which compared two different approaches of lifestyle intervention were not in the scope of this review. These were for example studies which assigned patients assigned to sports activities or diet management. Those studies compared the effects of specific sports or diets rather than investigate effects of the provider-led interventions.

Healthcare professionals were defined as those with extensive knowledge including university-level study leading to the award of a first degree or higher qualification [18], in most cases – physicians, nurses and pharmacists. Studies that included less than 10 patients were excluded as they would be more heterogeneous and a high possibility of selection bias in a small study could occur [19]. Interventions were defined as tailored when the contact with a healthcare professional was intensified or de-intensified, based on the patient’s blood pressure data.

### Outcome measures

Outcomes of the review were the difference in systolic and diastolic blood pressure between intervention and control groups, and the difference in the proportion of patients achieving BP control.

### Data collection and analysis

The systematic review protocol was created. Search keys for PubMed, Embase and Cinahl were built; additionally, the references of indicated papers were searched. The PubMed search key could be found in the Additional file 1.

The search was carried out on the 18th of May 2020.

Two researchers – I.T. and J.P. – independently reviewed titles, abstracts and full articles. Reviewers separately reviewed the extracted data on number of patients, the duration of intervention and follow-up, intervention components as well as baseline and follow-up blood pressure measurements and discussed the discrepancies. In case of discrepancies, cases were discussed with a third reviewer K.T. If any data required for the analyses was missing in the retrieved articles, the authors were contacted.

Two researchers – I.T. and T.F. – assessed the risk of bias using the Cochrane risk of bias tool [20]. Non-pharmacological interventions may introduce more biases as the participants and personnel cannot always be blinded due to the nature of the interventions. Baseline characteristics of included patients may have an impact on the overall assessment of biases as well. Nine criteria for the assessment of risk of bias were used, including random sequence generation, allocation concealment, similarity of baseline outcome measurements, similarity of baseline characteristics, blinding of outcome assessment, incomplete outcome data, contamination, selective reporting and other biases reported by the investigators. Biases were assigned to one of the three categories – low risk, unclear risk and high risk. Studies having 4 or more criteria scoring high risk/unclear risk were categorized as having an overall high risk of bias.

Meta-analyses by RevMan 5.4 using a random effects analysis model was performed. The mean difference in systolic and diastolic blood pressure between the intervention and the usual care group was calculated, as well as the percentage of patients who achieved BP control in the intervention and the usual care group. The odds ratio for BP control were determined.

To avoid unit-of-analysis errors for cluster-randomized controlled trials (cRCTs) in which incorrect statistical analyses were conducted, an approximate analysis based on inflating standard errors was performed. Before entering data into RevMan the standard error of the effect estimate (from an analysis that does not take clustering into account) was multiplied by the square root of the design effect. The design effect was determined as 1+ (M-1) ICC, where M is the average cluster size and ICC – the intracluster correlation coefficient. The common design effect across the intervention groups was assumed. If the ICC was not available in the published report, the mean ICC from other included cRCTs was used. Sensitivity analyses for RCT comparing the data as presented and taking the intracluster correlation coefficient into account was performed.

If the study included more than two intervention arms and no control or usual care group, the intervention closest to usual care was considered as usual care. If the studies had two treatment arms and a usual care group, only the treatment arm that had lifestyle modification or health promotion provided by health care professional was included into meta-analyses. This approach was chosen in line with recommendations by Cochrane to overcome a unit-of-analysis error [20].

Means and standard deviations from a relevant intervention or a usual care group were pooled. If standard deviation (SD) was not provided in the study report, it was calculated with the “Cochrane collaboration finding standard deviations calculator” from other standard errors or confidence intervals given. If the data was missing or clarification needed the authors were contacted. If it was not possible to obtain SD, the mean SD of the included studies was used.

Heterogeneity according to the approach described in the Cochrane Handbook was assessed [20]. The I^2^ statistic to assess heterogeneity was used, considering heterogeneity to be statistically significant if the *p* value was less than 0.05. The interpretation of the I^2^ statistic was followed, 0–30% might not be important, 30–60% may represent moderate heterogeneity, 50–75% may represent substantial heterogeneity and 75–100% represents considerable heterogeneity. Since it was assumed that clinical and methodological diversity within the studies may occur, it was agreed that I^2^ value between 30 and 60% represents moderate heterogeneity, and I^2^ over 60% represents substantial heterogeneity.

### Data synthesis and subgroup analysis

Intervention effects for dichotomous data were calculated as odds ratio with the 95% confidence intervals. For continuous data, the mean differences with the 95% confidence intervals were calculated. Extreme outliers were excluded from the analysis, a sensitivity analysis including the outliers was performed.

Several subgroup analyses were carried out as recommended [21]. Subgroup analyses based on place the outcome blood pressure was taken (home or office), the duration of an intervention (under 6 months or longer than 6 months), by the type of healthcare professional and the type of intervention (face to face vs via device and tailored vs not tailored intervention) were performed. In further subgroup analyses the effects in patients with baseline systolic blood pressure under and above 150 mmHg were investigated. Finally, the effects of interventions which included medication adherence tool or antihypertensive medications review were investigated.

## Results

### The description of studies

**Fig. 1:**
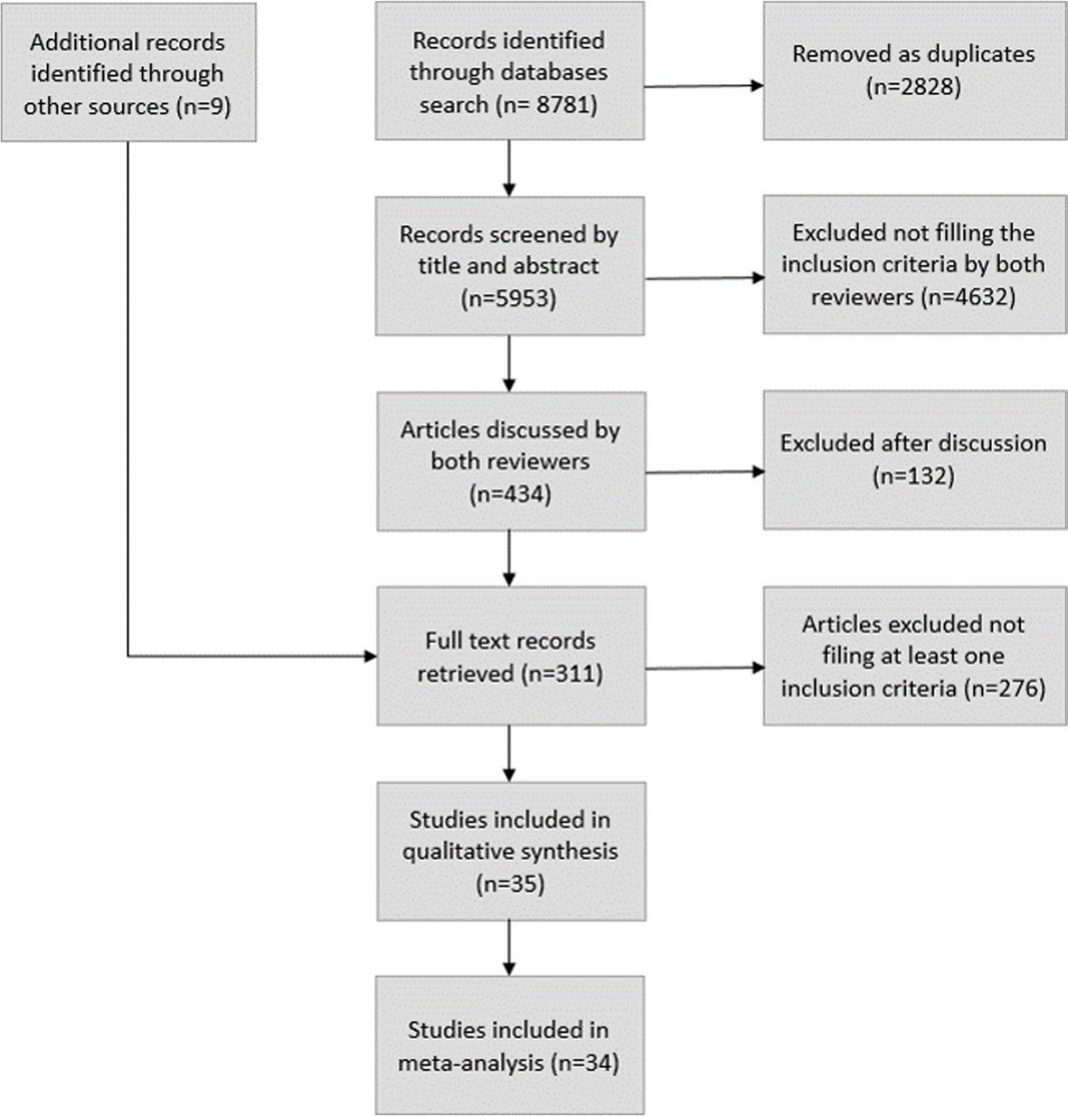
Flow diagram. Unique articles were identified from database searching. Articles were screened against eligibility criteria in two rounds by two independent authors. First screening by title and abstracts was carried out. Then after discussion, full text records were retrieved and screened against eligibility criteria independently and discussed again

Eight thousand seven hundred eighty-one articles were retrieved (Fig. 1).

Thirty-five clinical trials were included in the review. In the studies, 22,715 patients were randomized; the mean age was 58.1 years, 49% were female, 69% used antihypertensive medications. At least 23% of included patients were diagnosed with diabetes.

Of the included studies, 30 were individual randomized controlled trials and 5 were cluster randomized trials. Twenty studies were carried out in the United States [22–41], 4 in Europe [42–45], 2 in China [46, 47] 1 in Canada [48], Australia [49], Mexico [50], Taiwan [51], Pakistan [52], Thailand [53], South Africa [54], Japan [55] and India [56]. For 20 studies [24, 25, 27–29, 31–33, 38, 41, 43, 45–51, 53–56] a follow-up was completed within 6 months, in the remaining studies a follow-up was from 6 to 24 months (Table 1).

**Table 1:** Summary of included studies. Trial length, number of patients, blood pressure and demographic data pooled from the included studies

	Study design	Country	Trial length in month	Number of patients included	Proportion of patients under BP control %	Diastolic BP at recruitment, mmHg	Systolic BP at recruitment, mmHg	Patients with diabetes %	Female %	Mean age	Race	Proportion using antihypertensive medication, %
Artinian 2007	RCT	USA	8	387	0	> = 90	> = 140	25.83	64.34	59.65	100% African Americans	70.54
Borenstein 2003	RCT	USA	12	197	0	> = 90	> = 140	13	61	62	32% African Americans	N/A
Bosworth 2009	RCT	USA	24	636	73	di	di	36	66	61	49% African Americans	100
Bosworth 2011	RCT	USA	18	591	59	> 90	> 140	43	8	64	48% white	100
Brennan 2010	RCT	USA	12	638	14	di	di	26	67	55.7	100% African Americans	96
Garcia-Pena 2001	RCT	Mexico	6	718	0	> = 90	> = 160	26	64	70.6	N/A	77
Green 2008	RCT	USA	12	778	0	90–109	140–199	N/A	52.2	59.1	82,8% white	96
Hennessy 2006	cRCT	USA	6	10,696	53.29	di	di	25.48	56.95	62.3	48% Caucasian	66.77
Hunt 2004	RCT	USA	12	604	0	90–99	140–159	15	58	69	90% white	N/A
Hunt 2008	RCT	USA	12	463	0	> = 100	> = 160	25	65	68	N/A	N/A
Johnson 2011	RCT	USA	6	552	0	di	di	43	65	42	91% African Americans	96
Kastarinen 2002	RCT	Finland	24	715	N/A	90–109	140–179	N/A	53	54.3	N/A	52
Lang 1995	cRCT	France	18	129	0	> 90	> 140	N/A	5	43	N/A	19
Lee 2007	RCT	Taiwan	6	202	0	N/A	140–179	N/A	42	71.3	N/A	68
Little 2004	cRCT	USA	1	296	0	> 90	> 160	N/A	44	50	N/A	0
Ma 2014	RCT	China	6	120	N/A	di	di	N/A	50.83	58.76	N/A	100
Magid 2011	RCT	USA	6	338	0	> 90 (80^a^)	> 140 (130^a^)	54	35	65.4	65% white	100
Margolis 2013	cRCT	USA	18	450	0	> = 90	> = 140	19.11	44.7	61.1	81.8 white	73.8
McKenney 1973	RCT	USA	5	50	32	> 90	N/A	N/A	76	60	78% African American	N/A
McLean 2008	RCT	Canada	6	227	3	> 80	> 130	100	40	64.95	N/A	66.95
Mehos 2000	RCT	USA	6	36	0	90–109	140–179	19.44	69.44	58.8	77.77 Caucasian	100
Morgado 2011	RCT	Portugal	9	197	33	di	di	18	60	59.5	N/A	100
Roumie 2006	cRCT	USA	6	1827	0	> = 90	> 140	4.02	3	65	N/A	100
Saleem 2015	RCT	Pakistan	9	385	41.8	di	di	N/A	31.2	39	N/A	100
Sookaneknun 2004	RCT	Thailand	6	235	20.42	> = 90	> = 140	44	68	63.2	N/A	N/A
Stewart 2005	RCT	South Africa	6	83	N/A	di	di	N/A	N/A	N/A	N/A	N/A
Tobari 2010	RCT	Japan	6	132	38.63	90–109	140–179	N/A	34	61.65	N/A	94
Tonstad 2007	RCT	Norway	6	49	0	90–99	140–169	N/A	26.53	55	N/A	16.32
Vivian 2002	RCT	USA	6	56	0	> 90	> 140	51	0	64.75	77.4% African American	100
Wakefield 2011	RCT	USA	6	302	N/A	di	di	100	2	68	96% Caucasian	N/A
Wal 2013	RCT	India	6	142	0	> 90	> 140	40.19	55.88	60.06	N/A	N/A
Woollard 1995	RCT	Australia	4.5	166	N/A	di	di	N/A	46.57	58.3	N/A	100
Zabler 2018	RCT	China	6	59	N/A	di	di	N/A	63	53.73	100% African American	100
Zhu 2017	RCT	USA	4	134	0	> = 90	> 140	23.9	50.7	69	N/A	81
Zillich 2005	cRCT	USA	3	125	0	di	di	28	61	65	97.6% white	100

di - inclusion under diagnoses of hypertension, the range of inclusion BP not specified

*N/A* data not available

^a^ different inclusion blood pressure for diabetes patients

In all the studies, lifestyle modification was addressed. Two interventions were performed in community pharmacies [32, 48] one in pharmacy and primary care [53] all other studies were performed in primary care practices or outpatient centers of hospitals. In 16 studies the advice on dietary/sodium restriction was given [24, 28, 29, 34, 35, 37, 41, 42, 45, 46, 49, 50, 53–56]. Twenty studies addressed dietary/weight loss/dietary approaches to stop hypertension (DASH) [23, 24, 27, 31, 34–38, 40–42, 45, 47, 49, 50, 53–56]. Recommendations on alcohol consumption were given in 14 studies [24, 28, 34, 35, 41–43, 45–47, 49, 53, 55, 56]. Fourteen studies included smoking cessation [23, 24, 31, 34–36, 42, 45–47, 49, 53, 55, 56]. Exercise/physical activity was recommended in 20 studies [23, 24, 28, 29, 31, 34, 36, 37, 41, 42, 45–47, 49–51, 53–56]. Education on home blood pressure monitoring was provided and home BP devices were given in 13 studies [22, 24–26, 28, 31, 32, 34–37, 39, 55]. Seven studies included tailored interventions [25, 28, 31, 35, 37, 39, 46].

Healthcare professionals were specifically trained to give the intervention in 13 studies [22, 24, 29, 36–38, 41–43, 46, 47, 50, 52]. Thirteen studies included a medication review in addition to the lifestyle interventions. Those medication reviews were performed by the interventionist or if needed by a healthcare professional the participant was referred to. In those studies recommendations/changes or referrals for changes on antihypertensive medications or regimens were performed if needed, alongside with the lifestyle modification intervention [23, 25, 26, 28–30, 32, 35, 39, 46, 48, 53, 55]. In 11 studies adherence improvement was additionally implemented [23, 27, 28, 30, 41, 44, 47, 52–55]. Techniques such as pill count, patient diary, assessment with a questionnaire or data on adherence, obtained from the pharmacy and discussed with the patient, were used. In other studies, medication adherence was emphasized or was not mentioned at all. A summary of components of the interventions is provided in Table 2.

**Table 2:** Summary of components of the interventions. Intervention components pooled from included studies

	Intervention specialist	Outcome BP place	Tailored	Face-to face lifestyle management intervention	Specialist training	Components of the lifestyle intervention									Medication change referred or recommended
						Education on disease	Lifestyle advice not specified	Dietary (Na restriction)	Dietary/weight loss/DASH	Alcohol consumption	Smoking cessation	Exercise/physical activity	Adherence improvement strategies ^c^	Education on home blood pressure monitoring	
Artinian 2007	Nurse	Office	No	No	+		+							+	
Borenstein 2003	Team	Office	No	Yes			+		+		+	+	+		+
Bosworth 2009	Nurse	Office	No	No				+	+	+	+	+		+	
Bosworth 2011	Nurse	Office	Yes	No		+		+	+	+	+			+	+
Brennan 2010	Nurse	Home	No	No	+	+			+		+	+		+	
Garcia-Pena 2001	Nurse	Home	No	Yes	+	+	+	+	+			+			
Green 2008	Pharmacist	Office	Yes	No	+			+	+			+		+	
Hennessy 2006	Provider^a^	Office	No	No	+	+			+						
Hunt 2004	Primary care provider	Office	No	No		+	+		+					+	
Hunt 2008	Team	Office	Yes	Yes		+	+								+
Johnson 2011	Nurse	Office	No	Yes	+	+		+	+	+		+	+		
Kastarinen 2002	Nurse	Office	No	Yes	+			+	+	+	+	+			
Lang 1995	Physician	Office	No	Yes	+					+					
Lee 2007	Nurse	Office	No	Mixed								+			
Little 2004	Nurse	Home	No	Yes	+	+		+	+	+	+	+		+	
Ma 2014	Nurse	Office	No	Yes	+				+	+	+	+	+		
Magid 2011	Pharmacist	Office	Yes	No		+	+							+	+
Margolis 2013	Pharmacist	Office	No	Mixed		+	+							+	+
McKenney 1973	Pharmacist	Office	No	Yes		+			+				+		
McLean 2008	Team	Office	No	Yes		+	+								+
Mehos 2000	Pharmacist	Office	Yes	Mixed		+		+		+		+	+	+	+
Morgado 2011	Pharmacist	Office	No	yes		+	+						+		
Roumie 2006	Provider^b^	Office	No	No	+	+		+				+			+
Saleem 2015	Pharmacist	Office	No	Yes	+	+	+						+		
Sookaneknun 2004	Pharmacist	Office	No	Yes		+		+	+	+	+	+	+		+
Stewart 2005	Physician	Office	No	No				+	+			+	+		
Tobari 2010	Team	Home/office	No	Yes		+		+	+	+	+	+	+	+	+
Tonstad 2007	Nurse	Office	No	Yes				+	+	+	+	+			
Vivian 2002	Pharmacist	Office	No	Yes			+						+		+
Wakefield 2011	Nurse	nn	Yes	No			+		+		+	+		+	
Wal 2013	Pharmacist	Office	No	Yes		+		+	+	+	+	+			
Woollard 1995	Nurse	Office	No	Yes				+	+	+	+	+			
Zabler 2018	Nurse	Office	No	Mixed	+	+									
Zhu 2017	Nurse	Office	Yes	Mixed			+		+	+	+		+		+
Zillich 2005	Team	Office	No	Yes		+	+							+	+

Mixed intervention – first visit face to face, following visits by device

^a^ Physicians and nurse practitioners in family medicine, internal medicine or obstetrics-genecology

^b^ Attending physicians, resident physicians, nonphysician clinicians (nurse practitioner or physician assistant)

^c^ Adherence tools focused on patient used: pill count, dairy, assessment with questionnaire at time point visits or data on adherence obtained from data base or pharmacist and discussed with the patient at regular visits

The authors of five studies were contacted to obtain missing outcome data. Four responses were received. One study was excluded from the meta-analyses as the missing data could not be obtained. Thirty-four studies contained sufficient data to be included in the meta-analyses. Twenty two thousand four hundred nineteen patients with mean age of 58.4 years where randomized in those studies. Forty-nine point fourteen percent of patients were female, 69.97% used antihypertensive medications.

**Fig. 2:**
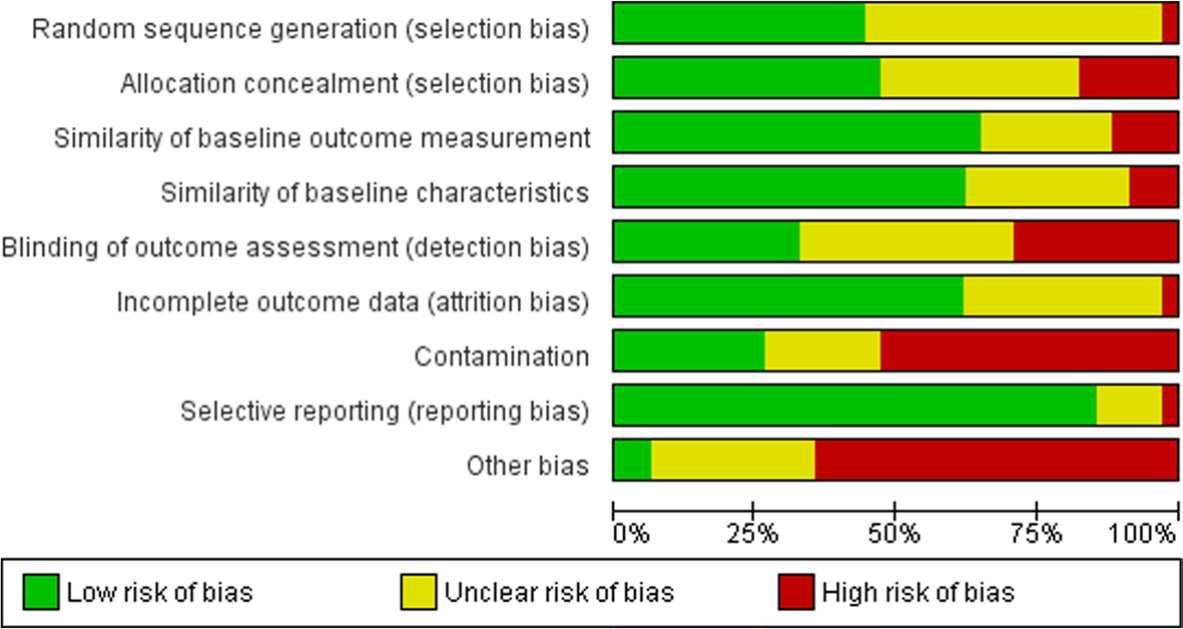
Overall assessment of the biases of included studies. Nine criteria for the assessment of risk of bias were used. Biases were assigned to one of the three categories – low risk, unclear risk and high risk

The risk of bias of the studies that were included into meta-analyses was evaluated. Nine studies were considered as low in the overall assessment of bias, 25 as high (Fig. 2).

Contamination was possible when healthcare professionals were allocated within the same clinic or practice; in this case the communication between intervention and control professionals as well as patients was possible. In the cluster randomized trials with the allocation by an institution or a practice, this risk was prevented. Other risks provided by the authors were also common. The other risks of biases reported were not taking clustering into account [26, 32, 43], self-selection biases [36] labelling, that might have caused the increased care by a physician [53] only highly motivated [42] or highly educated patients included [37] language literacy [39] real world barriers [41] etc.

### Systolic blood pressure

**Fig. 3:**
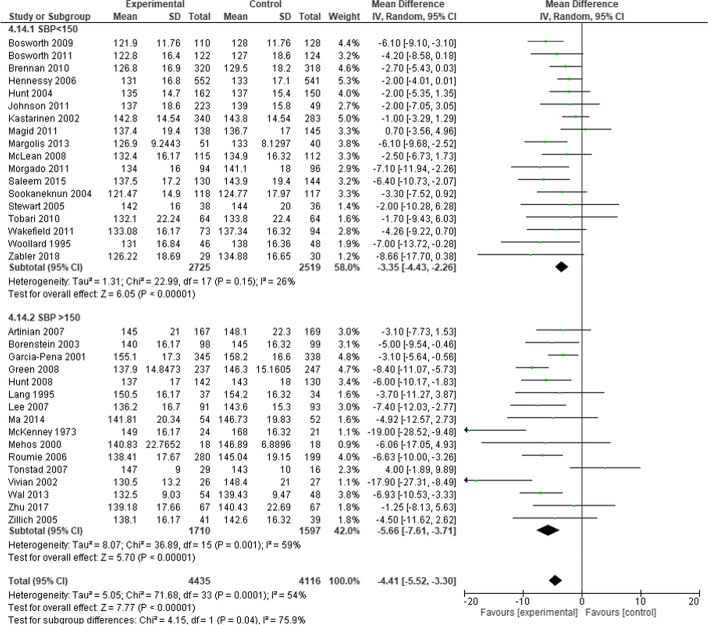
Subgroup analyses of the effect of interventions by baseline systolic blood pressure. Legend: Forest plot shows difference in systolic blood pressure change between patients with baseline systolic blood pressure over 150 mmHg versus patients with baseline systolic blood pressure lower than 150 mmHg; forest plot was created using RevMan 5.4; SBP – systolic blood pressure, CI – confidence interval, SD – standard deviation; small green squares represent difference in SBP reduction of individual RCTs, horizontal lines show 95% CI, black diamonds represent difference in SBP reduction within subgroup and total. Statistically significant difference was found comparing subgroups (*P* = 0.04)

The mean difference of the SBP between the intervention group and the usual care group was − 4.41 mmHg (95% CI, − 5.52 to − 3.30). Patients with SBP higher than 150 mmHg at baseline showed better response to intervention than patients with baseline SBP lower or equal to 150 mmHg. The mean difference SBP was − 5.66 mmHg (95% CI, − 7.61 to − 3.71) and − 3.35 mmHg (95% CI, − 4.43 to − 2.26) compared to the usual care group respectively (*P* = 0.04) (Fig. 3).

**Fig. 4:**
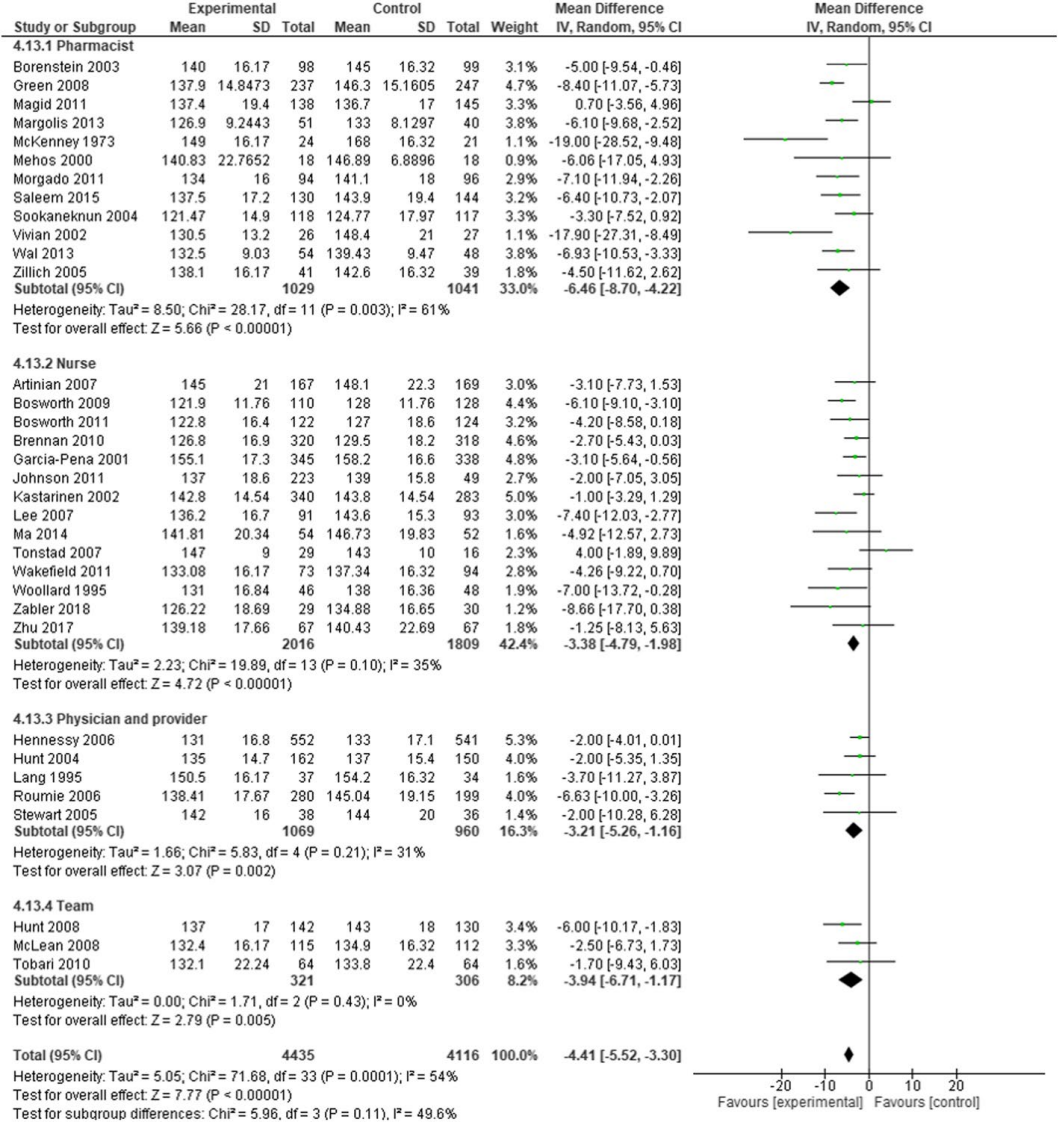
Subgroup analyses of the effect of interventions by different healthcare professionals. Forest plot shoes difference in systolic blood pressure change after interventions provided by different healthcare professionals individually (pharmacist, nurse or physician) or team, consisting of different healthcare professionals; forest plot was created using RevMan 5.4; CI – confidence interval, SD – standard deviation; small green squares represent difference in SBP reduction of individual RCTs, horizontal lines show 95% CI, black diamonds represent difference in SBP reduction within subgroup and total. No statistically significant difference was found between subgroups

There were no statistically significant differences in the mean reduction of SBP in the sub analysis by the type of a healthcare professional (Fig. 4).

There were also no statistically significant differences in the subgroup analyses by the duration of an intervention, the place of BP measurement, whether the interventions were tailored or not and whether the interventions contained a component addressing medication adherence (Table 3).

**Table 3:** The overview of subgroup analyses of the effect of interventions on SBP

Subgroups	Mean difference SBP mmHg (95% CI)		*P* value^1^
Duration of intervention	6 months and under	Over 6 months	
	−4.34 (−6.13 to −2.54)	−4.58 (−5.94 to −3.22)	*P* = 0.83
Place of blood pressure measurement	Home	Physician’s office/clinic	
	−2.85 (−4.66 to −1.04)	−4.50 (−5.74 to −3.26)	*P* = 0.14
Tailored intervention	Tailored	Not tailored	
	−4.40 (−7.19 to −1.60)	−4.37 (−5.58 to −3.15)	*P* = 0.99
Medication adherence improvement techniques	Used	Not used	
	−4.34 (− 5.91 to − 2.78)	−4.48 (− 6.09, − 2.88)	*P* = 0.90
Referral for medication change or adjustment	Included	Not included	
	−4.46 (−6.21 to − 2.71)	−4.40 (− 5.85 to − 2.96)	*P* = 0.96

^1^ *P* value shows if the difference in mean difference of SBP between subgroups is statistically significant. E.g., the subgroup difference between interventions that lasted up to 6 months and interventions that lasted over 6 months was not statistically significant, both interventions were effective

### Diastolic blood pressure

Measurements of diastolic blood pressure were provided in 32 studies. Analyses of intervention studies showed that DBP decreased by 1.66 mmHg (95% CI, − 2.44 to − 0.88), I^2^ = 63%. Heterogeneity of the included studies was too high to perform a subgroup meta-analysis.

### Blood pressure control

Seventeen studies provided data on the proportion of patients achieving BP control. Fifty-six percent of patients achieved BP control in an intervention group vs 44% in a usual care group, OR = 1.87 (95% CI, 1.51 to 2.31).

Subgroup analyses revealed that better BP control was achieved when the baseline systolic blood pressure was over 150 mmHg (*P* = 0.04). Subgroup analyses showed no differences in BP control when different intervention methods or components were used (Table 4). We could not perform subgroup analyses on BP control effect by the place of a BP measurement as the final BP was measured at home only in two studies [36, 55].

**Table 4:** The overview of the effect of interventions on BP control

Subgroups	OR (95% CI)		*P* value^2^
Duration of intervention	6 months and under	Over 6 months	
	1.64 (1.24 to 2.16)	2.14 (1.60 to 2.87)	*P* = 0.19
Baseline systolic blood pressure	Below 150 mmHg	Over 150 mmHg	
	1.51 [1.17, 1.96]	2.27 [1.83, 2.81]	*P* = 0.02
Healthcare provider	Single (pharmacist, nurse or physician alone)	Team	
	1.96 [1.47, 2.62]	1.81 [1.38, 2.36]	*P* = 0.68
Tailored intervention	Tailored	Not tailored	
	1.94 (1.16 to 3.25)	1.83 (1.44 to 2.32)	*P* = 0.84
Medication adherence improvement techniques	Used	Not used	
	2.35 (1.62 to 3.42)	1.65 (1.28 to 2.11)	*P* = 0.12
Referral for medication change or adjustment	Included	Not included	
	1.77 [1.41, 2.21]	2.06 [1.34, 3.16]	*P* = 0.54

^2^*P* value shows if the difference in OR between subgroups is statistically significant. E.g., patients with the initial SBP over 150 mmHg had a higher chance to achieve the BP under control than patients with the initial SBP assessment under 150 mmHg and the difference was statistically significant

## Discussion

### Summary

Interventions were effective and helped to achieve 4.41 mmHg decrease of SBP and 1.66 mmHg decrease of DBP. Statistically better SBP results and better BP control were achieved in the studies where baseline SBP was higher than 150 mmHg. Considering that nearly 70% of the patients were already taking medications, the additional SBP lowering by 5 mmHg might be the solution for a better hypertension control. Several reports support the clinical relevance of SBP reduction by 1–5 mmHg. SBP decrease of 1 mmHg reduces the risk of stroke by 5% [57]. Stamler et al. showed that the reduction of SBP by 5 mmHg is associated with a 7% lower risk of all-cause mortality, 9% lower risk of mortality due to coronary heart disease and 14% lower risk of mortality due to stroke [58].

In subgroup analysis, there were no differences in SBP and BP control between tailored and not tailored interventions, the different healthcare professionals performing the intervention and interventions including vs excluding medication review related components. This suggests that none of the features that were investigated had a preeminent impact, but rather the intervention itself. To further investigate the combined effects of lifestyle changes and medication change and adherence management on hypertension, individual patient data would be needed.

### Strengths and limitations

This study is the first systematic review on healthcare professional-led lifestyle interventions focusing on individual hypertensive patients.

Only two studies [25, 45] with blood pressure increase after an intervention were found. This might be that studies having a positive outcome were more likely to be published than those reporting negative results. However, sensitivity analysis with outliers showed that the estimate of the overall effect of interventions on BP was similar. As the heterogeneity was expected, the random effects analysis to allow for differences in the treatment effect from study to study was used. The potential sources of heterogeneity were explored by conducting subgroup analyses by the type of clinical trial method, the duration of intervention and the type of a healthcare professional performing the intervention. No difference in effect was found. Moreover, sensitivity analyses accounting for an overall assessment of bias and study size reported similar effects on BP. Causes of heterogeneity could be comorbidities, the number of medications or the age of the patients, but the reasons were not identified, as individual patient data would have been required. Differences in terms of interventions, data collection methods and setting may explain the heterogeneity as well.

### Comparison with existing literature

The study results support the idea that the modification of lifestyle is important for lowering blood pressure and managing cardiovascular risk. Results are in line with other systematic reviews on different types of interventions. Internet-based interventions showed to reduce SBP by 3.8 mmHg and DBP by 2.1 mmHg [59] and digital interventions to reduce SPB by 3.74 mmHg and DBP by 2.37 mmHg [60]. Self-monitoring of hypertension was associated with a significant decline in SBP by 3.96 mmHg and DBP by 1.85 mmHg [61].

All international guidelines recommend non-pharmacological approaches in the early stages of hypertension. In the ACC/AHA 2017 guidelines, interventions on lifestyle modifications have an even greater place in the management of hypertensive patients than in the European guidelines. ACC/AHA 2017 guidelines recommend lifestyle modifications to the patients having 130–139/80–89 mmHg blood pressure, reviewing the effects after 3 or 6 months [62]. Although traditionally interventions on lifestyle modifications are the domain of physicians or nurses, pharmacists could perform those interventions as well. This is in line with the developments to extend the traditional role of pharmacists [63, 64].

### Implications for research and practice

As the interventions were complex, the intervention component that had the maximum effect on blood pressure was not singled out. This may suggest the opportunity for future research. Individual patient data meta-analysis could explain the effects of interventions on different patients’ groups. Cost effectiveness studies could provide economic assessment of lifestyle interventions.

This systematic review shows that healthcare professional-led interventions on lifestyle modifications lowered elevated blood pressure and a higher percentage of patients had their blood pressure well controlled. The results suggest that healthcare professional-led interventions on lifestyle modifications should be implemented in daily practice. The barriers to implement such interventions include traditional practices and structures; sceptical, stereotypical attitudes from professionals; and factors related to the development of person-centered interventions [65].

## Conclusions

Healthcare professional-led interventions were effective. Patients achieved almost 5 mmHg decrease of SBP and more patients achieved BP control. The results suggest that efforts are needed for widespread implementation.

## Supplementary Information

**Additional file 1.** Search strategy. Search terms for PubMed

## Data Availability

The datasets used and/or analysed during the current study are available from the corresponding authors on reasonable request.

## Abbreviations

BP: Blood pressure
SBP: Systolic blood pressure
DBP: Diastolic blood pressure
CVD: Cardiovascular desease
CI: Confidence interval
RCT: Randomized clinical trial
cRCT: Cluster randomized clinical trial
ICC: Intracluster correlation coefficient
