# Supplementary material for: Healthcare professional-led interventions on lifestyle modifications for hypertensive patients – a systematic review and meta-analysis
Source: BMC Fam Pract. 2021 Apr 5;22:63. doi: 10.1186/s12875-021-01421-z (PMC8022420; doi:10.1186/s12875-021-01421-z)
Supplement: Supplementary file 1 — Additional file 1. Search strategy. Search terms for PubMed [file 12875_2021_1421_MOESM1_ESM.docx]

Search terms for PubMed

#1: "Hypertension"[MeSH] OR hypertension OR "blood pressure"[MeSH] OR blood pressure

#2: "Clinical Trial"[Publication Type] OR "Clinical Trials as Topic"[MeSH] OR clinical trial

#3: "Health Behavior"[MeSH] OR health behavior OR "Health Promotion"[MeSH] OR health promotion OR "Patient Education as Topic"[MeSH] OR patient education OR "Counseling"[MeSH] OR counseling OR coaching OR "Exercise"[MeSH] OR exercise OR "Behavior therapy" [MeSH] OR behavior therapy OR behavior change OR "Diet" [MeSH] OR diet OR “weight reduction programs"[MeSH] OR weight reduction OR motivational interviewing OR lifestyle interventions OR advice on lifestyle OR “Risk reduction behavior"[MeSH] OR risk reduction behavior OR Smoking Cessation OR smoking cessation OR "Drinking Behavior"[MeSH] OR drinking behavior OR alcohol reduction OR stress OR "Case management" [MeSH] OR Case management

#4: "Health Personnel"[MeSH] OR Health Personnel OR "Nurses"[MeSH] OR nurses OR nurse "Personnel, Hospital"[MeSH] OR personnel OR "Pharmacists"[MeSH] OR pharmacist OR "Physicians" [MeSH] OR physician OR “Health Occupations” [MeSH] OR "Physical Therapy Modalities"[MeSH] OR community health OR "Physical Therapy Modalities"[MeSH]

**→**#1 AND #2 AND #3 AND #4
